# Xiao-ai-ping injection adjunct with platinum-based chemotherapy for advanced non-small-cell lung cancer: a systematic review and meta-analysis

**DOI:** 10.1186/s12906-019-2795-y

**Published:** 2020-01-13

**Authors:** Fanchao Feng, Jingyi Huang, Zhichao Wang, Jiarui Zhang, Di Han, Qi Wu, Hailang He, Xianmei Zhou

**Affiliations:** 1 Department of Respiratory Medicine, Affiliated Hospital of Nanjing University of Chinese Medicine, Nanjing, 210029 People’s Republic of China; 2 Department of Respiratory Medicine, Jiangsu Province Hospital of Chinese Medicine, 155 Hanzhong Road, Nanjing, 210029 People’s Republic of China; 3 Department of Integrative Medicine and Neurobiology, State Key Laboratory of Medical Neurobiology, School of Basic Medical Sciences, Institutes of Brain Science, Brain Science Collaborative Innovation Center, Shanghai Medical College, Fudan University, Shanghai, 200032 People’s Republic of China; 4 Department of Physiology, Xu Zhou Medical University, Xu Zhou, 221009 People’s Republic of China

**Keywords:** Xiao-ai-ping injection, Platinum-based chemotherapy, Advanced non-small cell lung cancer, Objective tumor response, Adverse side effects, Meta-analysis

## Abstract

**Background:**

Xiao-ai-ping injection (XAPI), as patented Chinese medicine, has shown promising outcomes in non-small-cell lung cancer (NSCLC) patients. This meta-analysis investigated the efficacy and safety of XAPI in combination with platinum-based chemotherapy.

**Methods:**

A comprehensive literature search was conducted to identify relevant studies in Pubmed, EMBASE, the Cochrane Library, Chinese National Knowledge Infrastructure, Wangfang Database, VIP Database, and Chinese Biology Medical Database from the date of their inception to September 2018. The RevMan 5.3 software was applied to calculate the risk ratio (RR) and mean difference (MD) with 95% confidence interval (CI).

**Results:**

We included and analyzed 24 randomized controlled trials. The meta-analysis showed that XAPI adjunctive to platinum-based chemotherapy had better outcomes in objective tumor response rate (ORR) (RR: 1.27, 95% CI, 1.14–1.40); improved Karnofsky performance scores (KPS) (RR: 1.70, 95% CI, 1.48–1.95); reduction in occurrence of grade 3/4 leukopenia (RR: 0.49, 95% CI, 0.38–0.64), anemia (RR: 0.63, 95% CI, 0.46–0.87) and thrombocytopenia (RR: 0.53, 95% CI, 0.38–0.73), nausea and vomiting (RR: 0.57, 95% CI, 0.36–0.90); and enhanced immune function (CD8^+^ [MD: 4.96, 95% CI, 1.16–8.76] and CD4^+^/CD8^+^ [MD: 2.58, 95% CI, 1.69–3.47]). However, it did not increase dysregulated liver and kidney function, diarrhea, constipation, and fatigue. Subgroup analysis of ORR and KPS revealed that dosage, treatment duration, and methodological quality did not affect the outcome significantly.

**Conclusions:**

Our meta-analyses demonstrated that XAPI in combination with platinum-based chemotherapy had a better tumor response, improved the quality of life, attenuated adverse side effects, and enhanced immune function, which suggests that it might be used for advanced NSCLC. Moreover, low dosage (< 60 ml/d) and long-term treatment of XAPI might be a choice for advanced NSCLC patients.

## Background

Lung cancer is becoming a health burden in recent years, despite some advances in treatment in the past few decades [1]. It remains one of the leading causes of cancer-related death worldwide, with approximately 1.8 million new lung cancer cases and 1.6 million deaths per year [2]. Non-small-cell lung cancer (NSCLC) is the common subgroup, which accounts for approximately 85% of lung cancer cases [3] and most NSCLC patients present with advanced-stage, unresectable disease. Platinum-based chemotherapy is still the cornerstone for the treatment of advanced NSCLC [4], with response rates of 25 to 35% and 1-year survival rate of about 30 to 40% [5]. However, platinum-based dual chemotherapy might result in frequent adverse effects and prevent completion of the recommended period of treatment, which finally may affect the therapeutic efficacy [6]. Therefore, there is a need to develop new strategies to maximize tumor control and minimize adverse effects in patients with advanced NSCLC.

In China, many patented Chinese medicines are used as adjunctive therapy and have gained popularity because of their potential effects in NSCLC patients [7–11]. Xiao-ai-ping injection (XAPI) which is extracted from Marsdenia Tenacissima Caulis, has been shown to have valid antitumor effects [12–15], including in lung cancer. The major bioactive compounds in XAPI are steroids, including tenacigenin A and tenacigenin B [16]. Modern pharmacological studies have demonstrated that XAPI, could enhance the gefitinib efficacy [17], and overcome erlotinib and gefitinib cross-resistance [18] in NSCLC patients.

Recently, many trials have reported that XAPI in combination with platinum-based chemotherapy is superior in improving objective tumor response, decreasing the severe adverse side effects, improving the quality of life and ameliorating symptoms in patients with advanced NSCLC. However, in some Chinese clinical trials, it is uncertain whether there is robust evidence on the efficacy and safety of XAPI as an adjuvant therapy to platinum-based chemotherapy. Thus, we conducted a comprehensive systematic review on the efficacy and safety of XAPI as an adjunctive therapy for advanced NSCLC.

## Methods

### Data resources and searching strategies

To explore the efficacy and safety of XAPI on patients with advanced NSCLC, relevant electronic data sources, including PubMed, EMBASE, the Cochrane Library, Chinese National Knowledge Infrastructure (CNKI), Wanfang Database, VIP Database and Chinese Biology Medical Database (CBM) were searched for relevant articles from the date of their inception to December 2018. The language was restricted to Chinese and English. The search words were not limited to MeSH, and free words were also used as follows: (Neoplasm [Mesh] OR Lung Neoplasm [Mesh] OR Pulmonary Neoplasms OR Pulmonary Neoplasm OR Lung Cancer OR Thoracic Neoplasm OR Pulmonary Cancer OR Lung Carcinoma OR Pulmonary Carcinoma OR NSCLC OR Non-small Cell Lung Cancer) AND (Xiaoaiping OR *Marsdenia tenacissima* extract). This research was conducted by two independent reviewers (FCF and ZCW), and any discrepancies were resolved either by discussion or by a third author (HLH).

### Inclusion and exclusion criteria

Studies satisfying the following criteria were included: (1) Only clinical randomized controlled trials (RCTs); (2) Studies in which diagnosis of NSCLC had been verified by cytology or tissue biopsy. Furthermore, only patients with pathologically documented NSCLC of stage III or IV, according to the tumor-node-metastasis (TNM)-based staging of lung cancer were included; (3) studies where the treatment involved platinum-based chemotherapy with or without XAPI; and (4) Objective tumor response rate (ORR) and improvement of Karnofsky performance score (KPS) were regarded as the primary outcome measures. The adverse side effects and indicators of immune function were considered as secondary outcome measures.

All studies that did not meet the abovementioned inclusion criteria were excluded. In addition, studies were also excluded, if they were review articles, animal experiments, duplicated publications, inappropriate interventions, or they if did not present sufficient data.

### Outcome measures

The primary outcomes included ORR and improvement of KPS. ORR was considered as an indicator of antitumor effect and a surrogate for clinical benefit according to the Response Evaluation Criteria In Solid Tumors (RECIST) [19]. ORR was calculated by adding the complete response (CR) and partial response (PR). KPS was considered as an indicator of the quality of life in NSCLC patients after treatment [20]. An increase or decrease of 10-points in the KPS was regarded as an improvement or deterioration of quality of life, respectively. Hence, the improved performance status of patients was investigated and set as another primary outcome. In addition, secondary outcomes included reduction in grade 3/4 myelosuppression, nausea and vomiting, other adverse side effects (e.g. dysregulated liver and kidney function, diarrhea, constipation and fatigue), and immune function effects (the percentage of CD4^+^, CD8^+^, CD4^+^/CD8^+^).

### Data extraction and quality assessment

Data were extracted by two independent reviewers (FCF and JRZ) and the divergences were resolved by discussion with the third expert adjudicator (HLH). From the eligible articles, the following data were collected, (1) basic information such as the name of the first author, publication year, and language; (2) patient demographics, including the number of patients, performance status, and TNM stage in each group; and (3) detailed interventions, duration of treatment, and outcomes.

The quality of the included studies was assessed according to the criteria in the Cochrane Handbook for the Systematic Reviews of Interventions (version 5.1.0) [21]. The seven aspects evaluated in the report were random sequence generation, allocation concealment, blinding of participants and personnel, blinding of outcome assessment, incomplete outcome data, selecting reporting and other bias. Each aspect was categorized as a high, low, or uncertain risk. Furthermore, the quality of each trial was also assessed quantitatively with a scoring system from 0 to 14, which evaluated the following aspects including randomization, blinding, analysis, patient selection, comparability of groups at baseline, extent of follow-up, treatment protocol, cointerventions, and outcomes, as shown in Additional file 7: Table S1 [22]. This assessment strategy has been widely used in previous meta-analysis publications [23, 24]. Therefore, we adopted it in our study. The two independent reviewers (FCF and QW) performed the assessment and the disagreement was resolved by discussion or consulting the expert adjudicator (XMZ).

### Subgroup analysis

Pre-specified subgroup analyses were performed to determine the potential causes of heterogeneity in the effects of XAPI on the primary outcomes. It was hypothesized that the treatment efficacy of XAPI was higher in trials using: i) a higher XAPI dose (≥ 60 ml per day) rather than lower dose usage; ii) a longer duration (> 14 days) rather than shorter duration, and iii) those with a low methodological quality (as studies with a higher methodological quality tend to exhibit more modest treatment effect).

### Statistical analysis

Data were analyzed using RevMan 5.3. Pooled risk ratio (RR) and 95% confidence interval (95% CI) were used for statistical analysis of dichotomous variables, while statistical analysis for continuous variables was performed using mean difference (MD) and 95% CI. If the heterogeneity present in pooled studies (I^2^) was > 50% and/or *P* < 0.1, a random-effect model was used. Otherwise, a fix-effect model was applied. Outcomes were calculated using *P* values and *P* < 0.05 was considered statistically significant. Funnel plots were applied to evaluate the potential publication bias for primary outcomes if more than ten studies were included for a meta-analysis. Publication bias was further evaluated by Begg’s test using Stata 14.0 software.

## Results

### Data collection

**Fig. 1:**
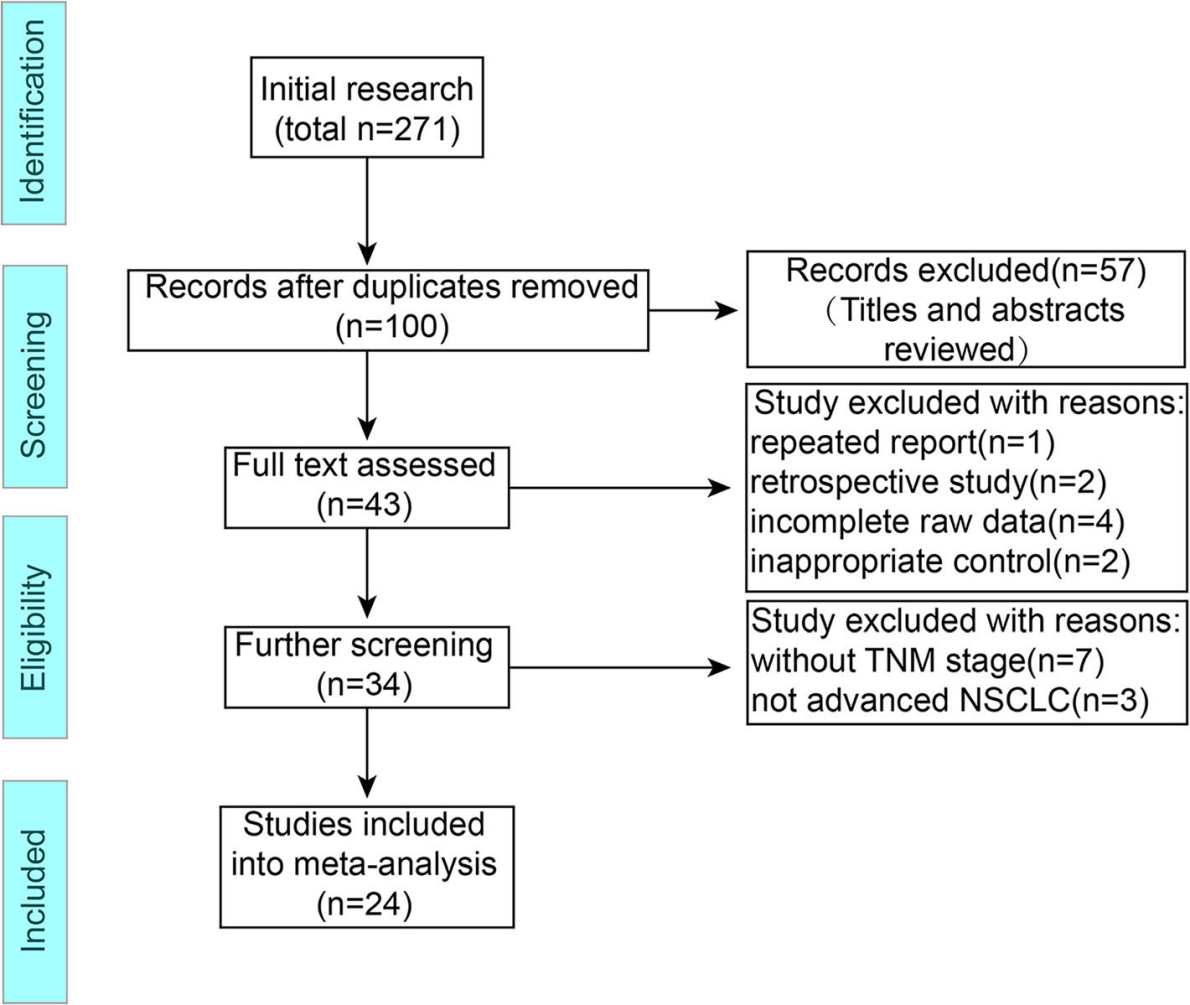
Flowchart of the trials selection process

Overall, 271 potentially relevant articles were identified from the seven databases in the initial search. After removing duplicated articles, the titles and abstracts of the remaining 100 studies were screened, and 57 records were removed owing to inappropriate intervention (*n* = 19), animal or in vitro experiments (*n* = 25), review articles (*n* = 8), combination with herbs extracts other than XAPI (*n* = 3), and treatment for malignant pleural effusions (n = 2). Subsequently, the full-text of 43 articles were screened, and eight were excluded because of repeated data, retrospective nature of the study, incomplete raw data, and inappropriate control group. Finally, 24 were included in the meta-analysis after removing studies that did not provide detailed TNM stage. A flow diagram of the selection process is presented in Fig. 1.

### Characteristics of the included studies

Twenty-four studies [25–48] published between 2007 and 2017, were included. These trials were all conducted in China. Overall 1725 patients with advanced NSCLC (863 who received XAPI in combination with platinum-based chemotherapy and 862 who received platinum-based chemotherapy alone) were identified and included in this meta-analysis. XAPI was administered intravenously, except in one study where it was administered intramuscularly [39]. The dosage of XAPI varied from 4 to 80 ml/d and the treatment duration ranged from one to four cycles. Among these researches, GP (Gemcitabine plus Cisplatin) [26–30, 39, 41, 44], TP (Taxol plus Cisplatin) [25, 31, 33–38, 46] and NP (Navelbine plus Cisplatin) [42, 43, 45, 47] regimens were the most common regimens. According to pathological TNM stages of NSCLC, all patients enrolled in the studies had stage III-IV disease. The demographic and clinical baseline characteristics of the included studies, such as the first author name, year of publication, case number, performance status, pathological TNM stage, interventions, and outcomes, are presented in Table 1.

**Table 1:** Summary of the characteristics of the included studies

Author/year	N		Physical status	TNM stage	Intervention		Duration (cycle)	Outcomes	Methods score
	T	C			T	C			
Ye L et al., 2017 [25]	30	30	NR	IIIb-IV	TP + XAPI (60 ml/d, d1–10)	TP	1	①②③④⑤⑥⑦	8
Hu XL et al., 2017 [26]	53	53	NR	IIIb-IV	GP + XAPI (20 ml/d, d1–15)	GP	4	①③④⑤⑦⑧⑨⑩	9
Gu N et al., 2016 [27]	39	39	NR	III-IV	GP + XAPI (22 ml/d, d1–22)	GP	1	①②	7
Liu JR et al., 2016 [28]	30	30	ZPS ≤ 3	IIIb-IV	GP + XAPI (40 ml/d, d1–14)	GP	2	①	7
Li XG et al., 2016 [29]	64	58	KPS > 70	IIIb-IV	GP + XAPI (80 ml/d, d1–8)	GP	4	①③④⑤	7
Li QL et al., 2016 [30]	36	36	KPS > 70	IIIb-IV	GP + XAPI (40–60 ml/d, d1–15)	GP	2	①②③④⑤⑥⑦⑧	9
Yao J, 2016 [31]	53	53	KPS ≥ 61	IIIb-IV	TP + XAPI (20 ml/d, d1–21)	TP	2	①③④⑤⑪	7
Song Y et al., 2016 [32]	40	40	NR	IIIb-IV	GP/NP + XAPI (40 ml/d, d1–15)	GP/NP	1	①③④⑤⑥⑨⑩	8
Li YL et al., 2015 [33]	33	32	KPS ≥ 60	IIIb-IV	TP + XAPI (60 ml/d, d1–15)	TP	2	①②③④⑤⑥⑨⑩⑪	8
Mei CR et al., 2015 [34]	30	33	KPS ≥ 60	IIIb-IV	TP + XAPI (20 ml/d, d1–14)	TP	2	①②③④⑤⑥⑦⑧	8
Shen LW et al., 2015 [35]	28	28	KPS ≥ 60	III-IV	TP + XAPI (40–60 ml/d, d1–15)	TP	2	①③④⑤⑪	8
Ai L et al., 2015 [36]	33	32	KPS ≥ 60	IIIb-IV	TP + XAPI (60 ml/d, d1–14)	TP	2	①②⑨	7
Shi L et al., 2015 [37]	26	26	NR	IIIb-IV	TP + XAPI (60 ml/d, d1–14)	TP	3	①	7
Xia GA, 2013 [38]	39	39	KPS ≥ 50	IIIb-IV	TP + XAPI (40 ml/d, d1–14)	TP	2	①②	7
Fang H et al., 2013 [39]	43	43	KPS > 70	IIIb-IV	GP + XAPI (4 ml/d, d1–8)	GP	3	①③④⑤⑥	9
Yang WQ et al., 2013 [40]	37	37	PS 0–2	IIIb-IV	DP + XAPI (60 ml/d, d1–28)	DP	2	①②③④⑤⑥⑦⑧	9
Zhang FY et al., 2011 [41]	24	24	KPS > 50	IIIb-IV	GP + XAPI (40–60 ml/d, d1–15)	GP	2	①②③④⑤⑫	8
Yang H et al., 2011 [42]	30	30	KPS ≥ 60	IIIb-IV	NP + XAPI (60 ml/d, d1–7)	NP	2	①②	7
Bai RL, 2010 [43]	36	36	KPS ≥ 60	IIIa-IV	NP + XAPI (60 ml/d, d1–15)	NP	1	①②⑫	7
Li J et al., 2009 [44]	38	38	KPS > 70	IIIb-IV	GP + XAPI (40 ml/d, d1–15)	GP	2	①②③④⑤⑥⑦⑧⑨	8
Wang K et al., 2009 [45]	28	28	KPS > 50	IIIb-IV	NP + XAPI (20–60 ml/d, d1–15)	NP	2	①②③④⑤⑫	8
Wang WY et al., 2009 [46]	27	29	KPS ≥ 70	IIIb-IV	TP + XAPI (80 ml/d, d1–7)	TP	2	①②⑫	7
Song CP, 2008 [47]	36	36	KPS ≥ 60	IIIa-IV	NP + XAPI (60 ml/d, d1–15)	NP	2	①②	7
Huang ZQ et al., 2007 [48]	30	32	KPS ≥ 50	IIIb-IV	GP/TC/NP + XAPI (60 ml/d, d1–15)	GP/TP/NP	2	①②③④⑤⑥⑦⑧⑩⑫	8

*NR* Not reported, *DP* Docetaxel plus Cisplatin, *GP* Gemcitabine plus Cisplatin, *NP* Navelbine plus Cisplatin, *TP* Taxol plus Cisplatin; outcomes ① ORR; ② KPS; ③ leukopenia; ④ thrombocytopenia; ⑤ anemia; ⑥ nausea and vomiting; ⑦ liver function; ⑧ kidney function; ⑨ diarrhea; ⑩ constipation; ⑪ fatigue; ⑫ immune function

### Methodological bias of the included studies

**Fig. 2:**
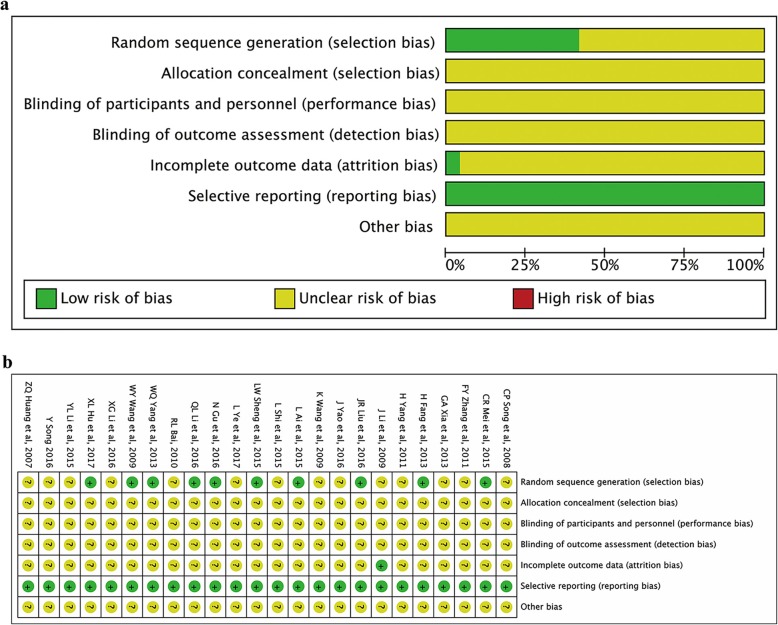
Risk of bias of the included studies. **a** Risk of bias graph (**b**) Risk of bias summary

The risk of bias for each study was assessed according to the Cochrane Handbook for Systematic Reviews of Interventions 5.1.0. All 24 studies declared the randomization, but only eleven [26–28, 30, 34–36, 39, 40, 44, 46] provided the details of the randomization process. Allocation concealment or blinding procedure was not mentioned. Only one study reported the drop-out data. No selective reporting bias was observed among all the trials. The presence of other bias was unclear. The detailed delineation of methodological quality of the included studies is summarized in Fig. 2. According to the score system we used in this meta-analysis, the mean methodologic score of the individual trials was 7.7 and median was 7.5 (range 7–9). The individual methodologic scores of each trial are available in Table 1.

### Primary outcomes

#### Objective tumor response rate

**Fig. 3:**
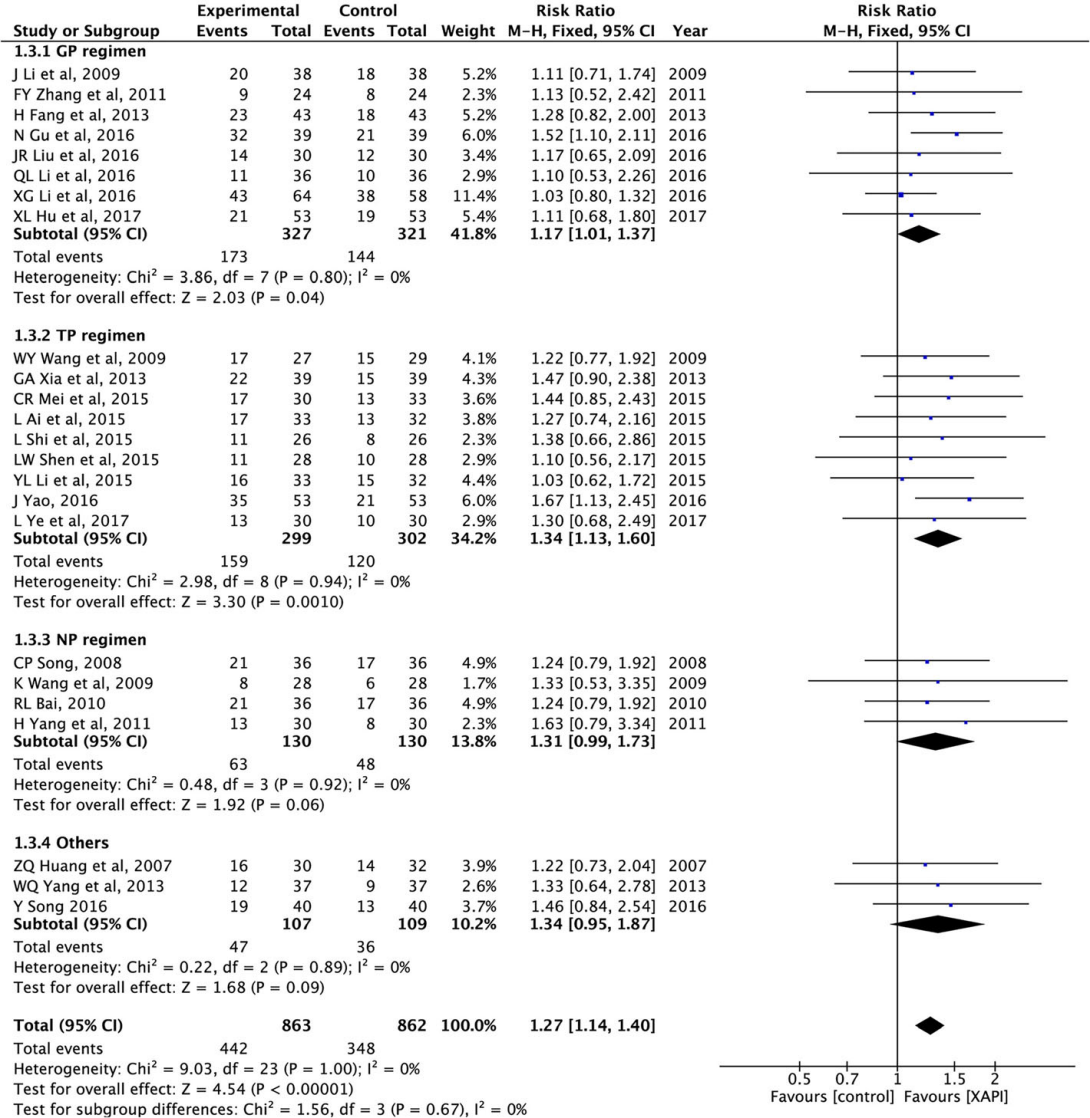
Forest plots showing objective tumor response rate (ORR)

ORR was reported in all 24 studies, for a total of 1725 patients [25–48]. As shown in Fig. 3, there was no significant heterogeneity (I^2^ = 0%, *p* = 1.00) among the studies; thus, the fix-effect model was used to analyze the data. The results showed that the combination of XAPI and platinum-based chemotherapy significantly increased the number of patients who showed CR or PR, compared with platinum-based chemotherapy alone (RR: 1.27, 95% CI, 1.14–1.40, *p* < 0.00001). In the subgroup analysis, the pooled RR showed significance in GP (RR: 1.17, 95% CI, 1.01–1.37, *p* = 0.04) and TP (RR: 1.34, 95% CI, 1.13–1.60, *p* = 0.001).

#### Quality of life

**Fig. 4:**
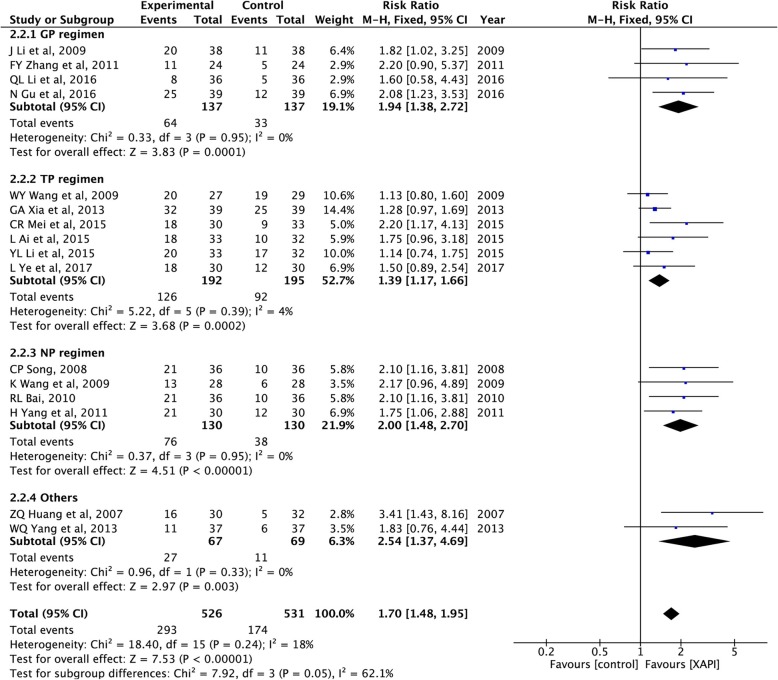
Forest plots showing Karnofsky Performance Status (KPS)

As shown in Figs. 4, 16 of the 24 included studies [25, 27, 30, 33, 34, 36, 38, 40–48] had evaluated the changes in KPS. As there was no evidence of significant heterogeneity between the studies (I^2^ = 18%, *p* = 0.24), the fix-effect model was selected for analysis. The pooled analysis revealed that a significant difference (RR: 1.70, 95% CI, 1.48–1.95, *P* < 0.00001) existed between the XAPI combination group and control group, suggesting XAPI combined with platinum-based chemotherapy could improve the quality of life. In the subgroup analysis, the pooled RR showed significance in GP (RR: 1.94, 95% CI, 1.38–2.72, *P* = 0.0001), TP (RR: 1.39, 95% CI, 1.17–1.66, *P* = 0.0002), NP (RR: 2.00, 95% CI, 1.48–2.70, P < 0.00001) and other regimen (RR: 2.54, 95% CI, 1.37–4.69, *P* = 0.003).

### Secondary outcomes

#### Reduction in grade 3/4 myelosuppression

**Fig. 5:**
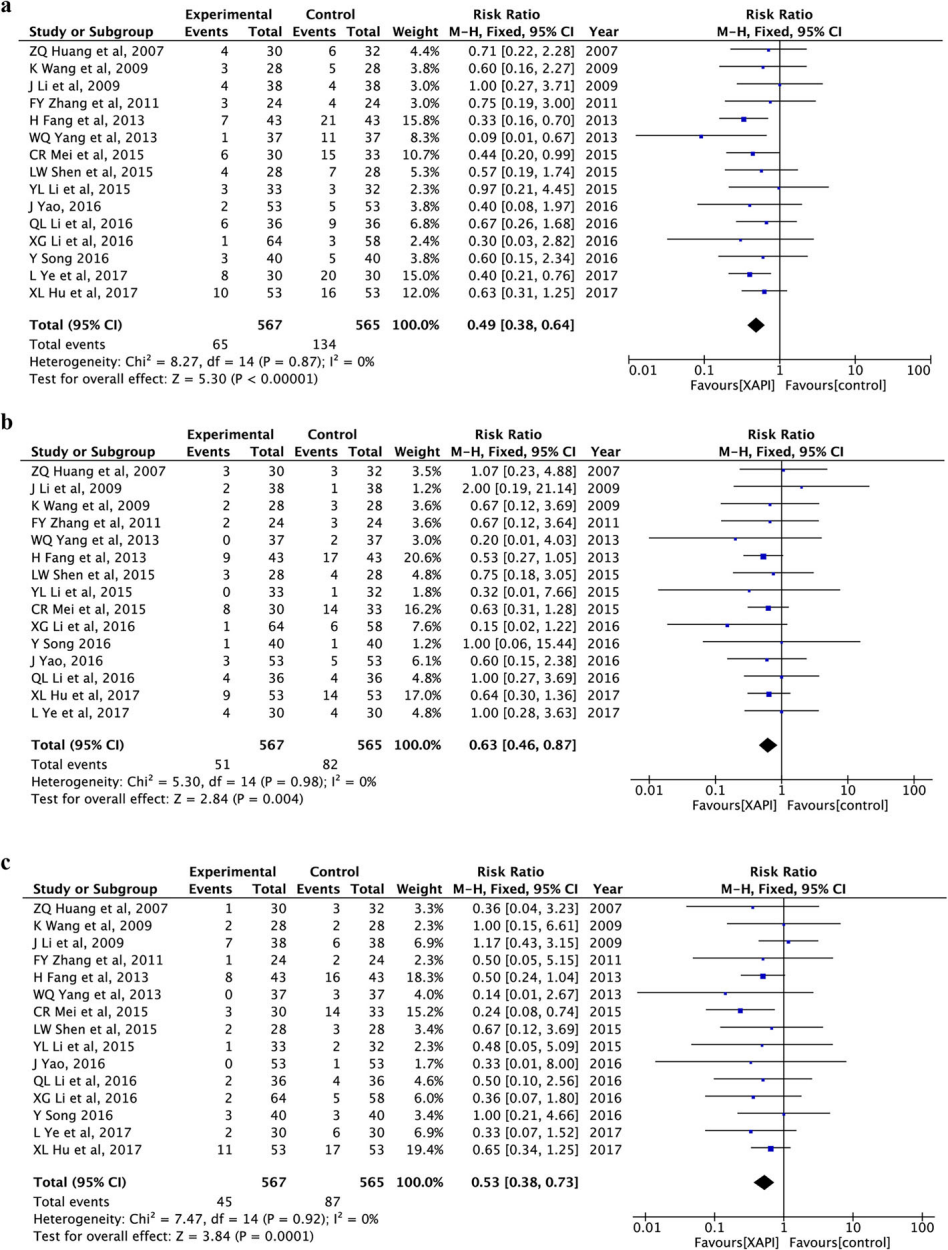
Forest plots showing reduction in grade 3/4 myelosuppression, including (**a**) leukopenia, (**b**) anemia, (**c**) thrombocytopenia

The incidence of leukopenia, anemia and thrombocy-topenia was reported in 15 trials [25, 26, 29–35, 39–41, 44, 45, 48], involving 1132 patients. The fix-effect model was used because of little heterogeneity (I^2^ = 0%, *p* > 0.1). Patients who received XAPI in combination with platinum-based chemotherapy had lesser occurrence of grade 3/4 leukopenia (RR: 0.49, 95% CI, 0.38–0.64, *p* < 0.00001; Fig. 5a), anemia (RR: 0.63, 95% CI, 0.46–0.87, *p* = 0.004; Fig. 5b) and thrombocytopenia (RR: 0.53, 95% CI, 0.38–0.73, *p* = 0.0001; Fig. 5c) than those who received platinum-based chemotherapy alone.

#### Reduction in grade 3/4 nausea and vomiting

**Fig. 6:**
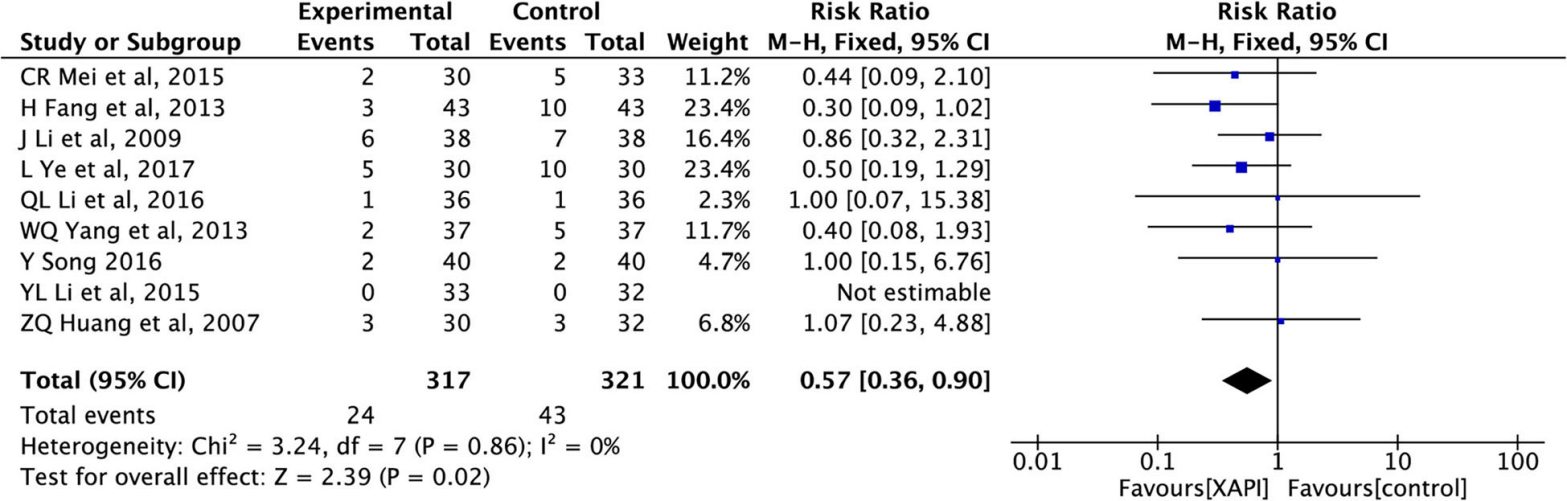
Forest plot showing reduction in grade 3/4 nausea and vomiting

The grade 3/4 nausea and vomiting were reported in nine trials [25, 30, 32–34, 39, 40, 44, 48], including 638 patients. The heterogeneity test showed a homogeneous group of studies (I^2^ = 0%, *p* = 0.86), and therefore, the fixed effect model was applied. The outcome revealed that XAPI could reduce the incidence of nausea and vomiting compared to the control group (RR: 0.57, 95% CI, 0.36–0.90, *p* = 0.02), as shown in Fig. 6.

#### Other adverse side effects

**Fig. 7:**
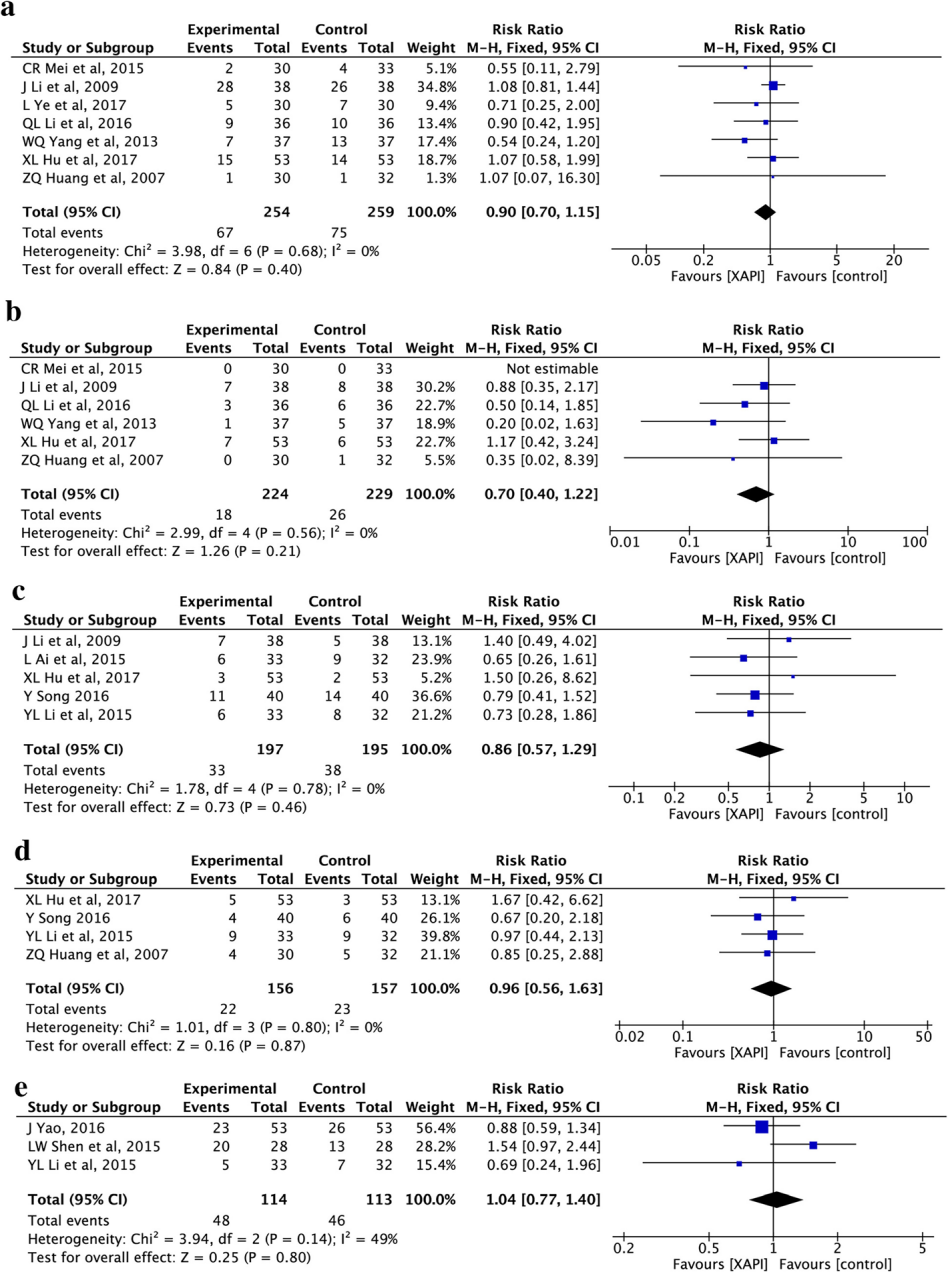
Forest plot showing other adverse side effects, including dysregulated (**a**) liver and (**b**) kidney function, (**c**) diarrhea, (**d**) constipation and (**e**) fatigue

Dysregulated liver and kidney function, diarrhea, constipation and fatigue were also reported in seven [25, 26, 30, 34, 40, 44, 48], six [26, 30, 34, 40, 44, 48], five [26, 32, 33, 36, 44], four [26, 32, 33, 48] and three [31, 33, 35] trials, respectively. The fix-effect model was used due to little heterogeneity. The results showed that XAPI did not cause an increase in the dysregulated liver (RR: 0.90, 95% CI, 0.70–1.15, *P* = 0.40; Fig. 7a) and kidney function (RR: 0.70, 95% CI, 0.40–1.22, *p* = 0.21; Fig. 7b), diarrhea (RR: 0.86, 95% CI, 0.57–1.29, *p* = 0.46; Fig. 7c), constipation (RR: 0.96, 95% CI, 0.56–1.63, *p* = 0.87; Fig. 7d) and fatigue (RR: 1.04, 95% CI, 0.77–1.40, *p* = 0.80; Fig. 7e).

#### Immune function

**Fig. 8:**
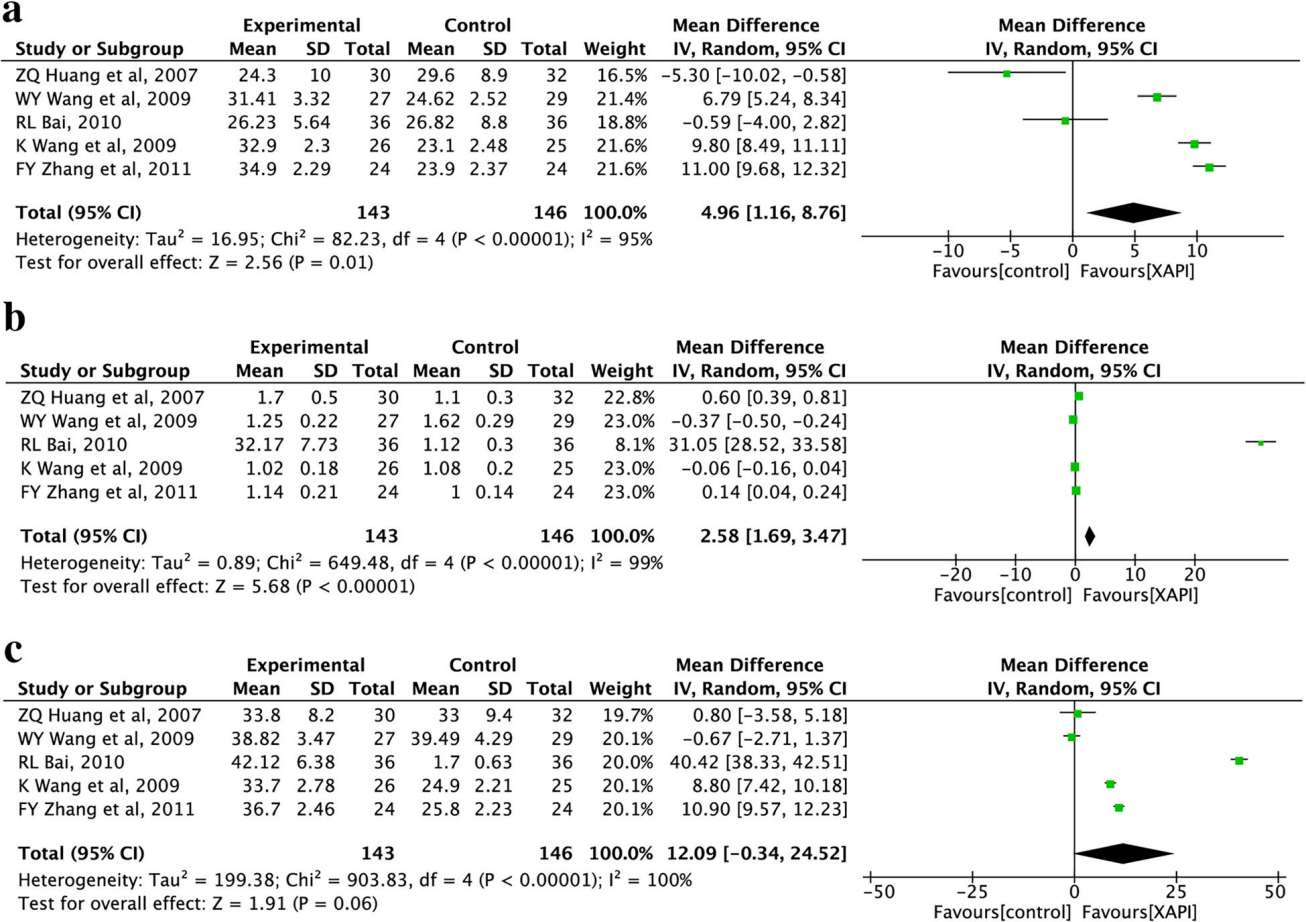
Forest plots showing comparison of immune function in Xiao-ai-ping injection plus chemotherapy and chemotherapy alone. **a** CD8^+^ T cell, **b** percentage of CD4^+^/CD8^+^, **c** CD4^+^ T cell

Five trials [41, 43, 45, 46, 48], including 289 patients, reported the percentage of CD4+, CD8+, and CD4+/CD8+. Due to the high heterogeneity, the random-effect model was used to calculate the MD and 95% CI. Meta-analysis indicated that there was a statistically significant difference between the two groups for CD8^+^ (MD: 4.96, 95% CI, 1.16–8.76, *p* = 0.01; Fig. 8a) and CD4^+^/CD8^+^ (MD: 2.58, 95% CI, 1.69–3.47, *p* < 0.00001; Fig. 8b), but not for CD4^+^ (MD: 12.09, 95% CI, − 0.34-24.52, *p* = 0.06; Fig. 8c), which indicated that XAPI combined with chemotherapy could enhance the immune function in NSCLC patients to some extent.

### Subgroup analysis of ORR

#### Low vs. high dose of XAPI

**Fig. 9:**
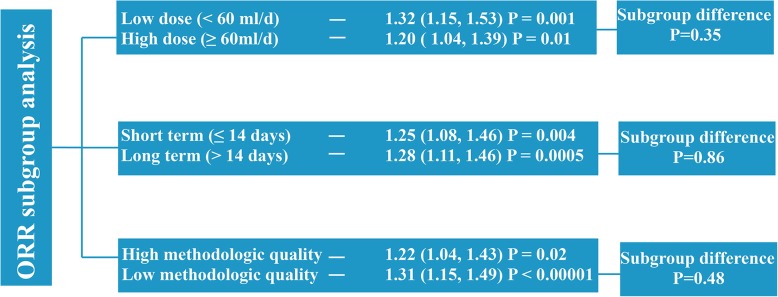
Summary of subgroup analysis of effects of Xiao-ai-ping-injection (XAPI) on objective tumor response rate (ORR) in patients with advanced NSCLC

Among the 24 studies that evaluated the ORR, 13 studies [26–28, 31, 32, 34, 35, 38, 39, 41, 44, 45] and 11 studies [25, 29, 33, 36, 37, 40, 42, 43, 46–48] reported the use of low (< 60 mg/d) and high (≥ 60 mg/d) dose of XAPI, respectively. The XAPI dose had no significant influence on the ORR, as shown in Fig. 9 and Additional file 1: Figure S1.

#### Short vs. long duration of treatment

A total of ten studies [25, 28, 29, 34, 36–39, 42, 46] and 14 studies [26, 27, 30–33, 35, 40, 41, 43–45, 47, 48] reported the administration of short (≤ 14 days in one treatment cycle) term (RR: 1.25, 95% CI, 1.08–1.46, *p* = 0.004) and long (> 14 days in one treatment cycle) term (RR: 1.28, 95% CI, 1.11–1.46, *p* = 0.0005) XAPI therapy. No significant difference was found in the outcomes between the two groups, as shown in Fig. 9 and Additional file 2: Figure S2.

#### Effect of the study quality on the outcomes

The mean of the methodological score was 7.7 and this was regarded as the cut-off score for categorizing the studies into high-(> 7.7) and low-(< 7.7) quality subgroups. Among the 24 studies that evaluated the ORR, 13 [25, 26, 30, 32–35, 39–41, 44, 45, 48] were included in the high-quality subgroup, while 11 [27–29, 31, 36–38, 42, 43, 46, 47] were included in the low-quality of subgroup. Although the treatment effects of XAPI did not show any significant difference between the two subgroups, low-methodological subgroup trials achieved a better outcome (RR: 1.27, 95% CI, 1.14–1.40, *p* < 0.0001) than the high-methodological subgroup (RR: 1.22, 95% CI, 1.04–1.43, *p* = 0.02). (Fig. 9 and Additional file 3: Figure S3).

### Subgroup analysis of KPS

#### Low vs. high dose of XAPI

**Fig. 10:**
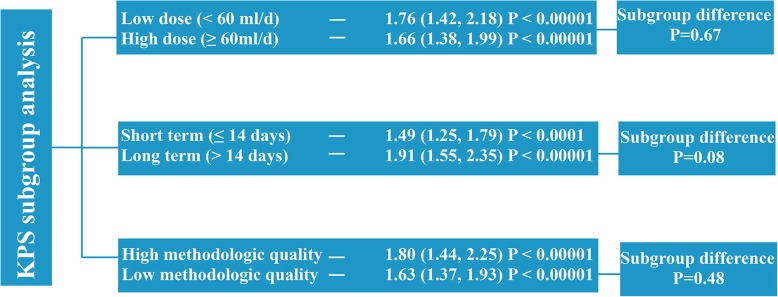
Summary of subgroup analysis of effects of Xiao-ai-ping-injection (XAPI) on Karnofsky Performance Status (KPS) in patients with advanced NSCLC

Among the 16 studies that evaluated the KPS, seven studies [27, 30, 34, 38, 41, 44, 45] and nine studies [25, 33, 36, 40, 42, 43, 46–48] reported the use of low (< 60 mg/d) and high (≥ 60 mg/d) dose of XAPI, respectively. The XAPI dose had no significant influence on the KPS (Fig. 10 and Additional file 4: Figure S4).

#### Short vs. long duration of treatment

A total of six studies [25, 34, 36, 38, 42, 46] and ten studies [27, 30, 33, 40, 41, 43–45, 47, 48] reported the administration of short (≤ 14 days in one treatment cycle) and long (> 14 days in one treatment cycle) term XAPI therapy. The duration of treatment did not influence the efficacy of XAPI significantly. However, the long-term subgroup showed a better outcome (RR: 1.91, 95% CI, 1.55–2.35, *p* < 0.00001) than the short-term subgroup (RR: 1.49, 95% CI, 1.25–1.79, p < 0.0001) (Fig. 10 and Additional file 5: Figure S5).

#### Effect of the study quality on the outcomes

The studies reporting on the KPS were also divided into a high-quality [25, 30, 33, 34, 40, 41, 44, 45, 48] and low-quality [27, 36, 38, 42, 43, 46, 47] subgroups, and no significant differences were observed between the two groups, as shown in Fig. 10 and Additional file 6: Figure S6.

### Publication bias

**Fig. 11:**
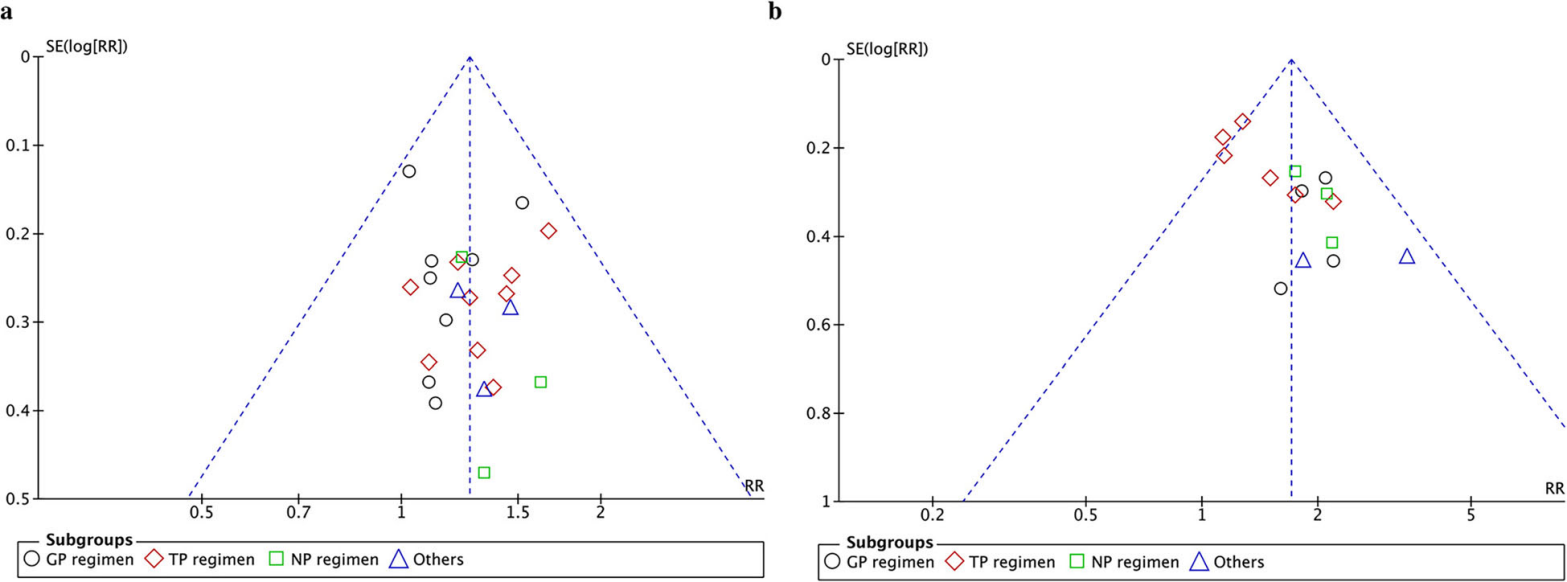
Funnel plots for assessing publication bias for (**a**) objective tumor response rate (ORR) and (**b**) improvement in Karnofsky Performance Status (KPS)

To identify the publication bias among the eligible studies, funnel plots and Begg’s test were utilized. As shown in Fig. 11, the funnel plots were asymmetric for ORR and improved KPS, suggesting potential publication bias. Begg’s test further confirmed that publication bias did not exist, as the *P* value for objective tumor response and improvement of KPS were 0.843 and 0.087, respectively.

## Discussion

In China, many patented Chinese medicines have been clinically integrated with standard chemotherapy in cancer patients for many years [49, 50]. However, their effectiveness is still a controversy worldwide due to the lack of systematic reviews summarizing the present evidence. *Marsdenia tenacissima* is known as “Tong-guan-Teng” or “Tong-guang-san” in Traditional Chinese Medicine (TCM) and its use was first recorded in ‘Dian Nan Ben Cao’ compiled by M-Lan dating back to Ming Dynasty. Its dried stems have been used for treating several kinds of diseases such as cancer, pneumonia, and tonsillitis. The extract of *Marsdenia tenacissima* has been available in China as a patented Chinese medicine named Xiao-ai-ping and is widely used for several cancers including NSCLC [51]. According to TCM, it is capable of relieving cough and asthma, eliminating phlegm, purging heat and has detoxifying effects. Modern pharmacology has identified polyoxypregnanes as the main effective ingredient, which can modulate P-glycoprotein and reverse multiple drug resistance in cancer [52]. XAPI could target apoptosis and autophagy leading to the death of NSCLC cells death [53]. Therefore, XAPI might enhance the efficacy of platinum-based chemotherapy. In addition, polysaccharide, another active ingredient of XAPI, can regulate the immune function and suppress tumor growth in mice [54]. Furthermore, XAPI can inhibit the migration of A549 cells [55], and serve as a potential anti-angiogenesis agent [56]. Taken together, XAPI might enhance the clinical efficacy of chemotherapeutic regimens by overcoming drug resistance, immunomodulation, and prevention of metastasis and tumor angiogenesis.

In the present meta-analysis, 1797 patients involved in 24 RCTs, were evaluated for the efficacy of XAPI in combination with platinum-based chemotherapy. The results of this meta-analysis showed that XAPI plus platinum-based chemotherapy was associated with better outcomes than platinum-based chemotherapy alone, as evidenced by higher ORR and improvement in KPS. In our subgroup analysis of different regimens, XAPI showed a relatively high ORR in the TP and GP regimens, which suggested that XAPI might play a synergistic effect in these subgroups. In addition, XAPI could improve the quality of life for NSCLC patients in all the subgroups, by increasing KPS. These results were encouraging and demonstrated that XAPI not only locally inhibit tumor growth but also improve the overall quality of life in NSCLC patients.

Adverse side effects are inevitable in most patients with advanced NSCLC receiving platinum-based chemotherapy, the severity of which can lead to treatment cessation in some cases. Myelosuppression and gastrointestinal reactions are the common platinum-based side effects. Study of the data showed, leukopenia, thrombocytopenia, anemia, nausea, and vomiting were the common grade 3/4 adverse side effects and our meta-analysis results revealed a significant reduction in their occurrence when XAPI was added to the platinum-based chemotherapy. Moreover, other side effects including dysregulated liver and kidney function, diarrhea, constipation, and fatigue, were also considered in this meta-analysis. The results showed that XAPI combined with chemotherapy did not increase the toxicity in the liver and kidney, which suggests that XAPI did not bring any metabolic burden to these organs. XAPI also did not cause more events in diarrhea, constipation, and fatigue, when combined with chemotherapy. Therefore, our results demonstrated that XAPI add-on platinum-based chemotherapy could decrease the platinum-based drugs induced severe myelosuppression and, nausea and vomiting but did not increase other side effects, supporting the efficacy and safety of XAPI.

In addition, immune function plays a vital role in lung cancer. Evidence showed that XAPI could improve the immune function to exert antitumor effects in mice [54]. Hence, the data associated with immune function were pooled into this meta-analysis to determine whether XAPI could potentiate the cellular immunity of advanced NSCLC patients. Though a small number of trials reported on these, the results revealed the percentages of CD8^+^ and CD4^+^/CD8^+^ cells were increased after treatment with XAPI, but not of CD4^+^ T cells. Collectively, these results illustrated that XAPI might exert anti-tumor effects by regulating immune function.

Furthermore, to address the potential heterogeneity in the primary outcomes, a hypothesis-based subgroup analysis of ORR was performed. The result showed that patients administrated with a low dose of XAPI exhibited better outcomes. It also suggested that doses under 60 ml/d might be the appropriate dose for NSCLC patients. The present meta-analysis also indicated that patients who received long duration treatment might have a higher ORR, though without significant subgroup difference (*p* = 0.86). Due to limitations in the methodological quality, subgroup analysis revealed better outcomes in studies with low methodological quality than those with high methodological quality. In the subgroup analysis of the KPS, the results were similar to ORR. We found that treatment dose and methodological quality did not influence the KPS. However, the results also demonstrated that advanced NSCLC patients who received long term treatment had a better outcome than those who received short term treatment. Collectively, this present meta-analysis demonstrated the efficacy of XAPI as an adjunctive therapy for advanced NSCLC.

Of note, there were some shortcomings in this meta-analysis. First, all the included studies were susceptible to some bias due to following reasons: flaws in the methodology, since specific method and procedure of the trials were unclear and obscure; dropouts and withdrawals that could not be extracted from the trials; lack of description of the details of randomization and concealment of allocation and blinding, although all the trials referred to randomization; no information on whether the syndrome differentiation based on TCM theory was taken into consideration in diagnostic procedures. Hence, the selection bias, performance bias, and detection bias were likely to be present in this meta-analysis. Second, our results only exhibited the overall efficacy and adverse side effects of XAPI plus platinum-based chemotherapy when compared with platinum-based chemotherapy alone. However, XAPI related efficacy and adverse side effects could not be uncovered in NSCLC patients. Further, there was a lack of information on long-term follow-up to evaluate the efficacy and safety of XAPI. Third, all trials were conducted in China and published in Chinese. Therefore, whether it could be beneficial to a wide population is uncertain. Fourth, the results of our meta-analysis might be affected by various therapeutic dosages and durations of treatment. Finally, we did not contact the authors for detailed information and failed to search for unpublished trials. Altogether, due to the insufficient methodological quality of the included trials, the potential benefits of XAPI for NSCLC patients need to be further verified through high-quality RCTs with rigorous methodology and adequate assessment of the safety profiles of the interventions.

## Conclusions

In summary, this meta-analysis suggested that XAPI in combination with platinum-based chemotherapy had a better tumor response, improved the quality of life, attenuated adverse side effects, and enhanced immune function in NSCLC patients. In addition, low dosage and long-term treatment of XAPI might be a choice for advanced NSCLC patients However, the results should be interpreted with caution, due to possible methodological flaws. More well-designed trials with bigger sample sizes and multicenter studies are urgently warranted to confirm this conclusion.

## Supplementary information

**Additional file 1: Figure S1.** Subgroup analysis of effects of Xiao-ai-ping-injection (XAPI) on the objective tumor response rate (ORR) in patients with advanced NSCLC according to the different dosages of the analyzed studies.

**Additional file 2: Figure S2.** Subgroup analysis of effects of Xiao-ai-ping-injection (XAPI) on the objective tumor response rate (ORR) in patients with advanced NSCLC according to the treatment duration of the analyzed studies.

**Additional file 3: Figure S3.** Subgroup analysis of effects of Xiao-ai-ping-injection (XAPI) on the objective tumor response rate (ORR) in patients with advanced NSCLC according to the methodological quality of the analyzed studies.

**Additional file 4: Figure S4.** Subgroup analysis of effects of Xiao-ai-ping-injection (XAPI) on the Karnofsky Performance Status (KPS) in patients with advanced NSCLC according to the different dosages of the analyzed studies.

**Additional file 5: Figure S5.** Subgroup analysis of effects of Xiao-ai-ping-injection (XAPI) on the Karnofsky Performance Status (KPS) in patients with advanced NSCLC according to the treatment duration of the analyzed studies.

**Additional file 6: Figure S6.** Subgroup analysis of effects of Xiao-ai-ping-injection (XAPI) on the Karnofsky Performance Status (KPS) in patients with advanced NSCLC according to the methodological quality of the analyzed studies.

**Additional file 7: Table S1** Criteria used to assess methodological score.

## Data Availability

Specific study data are available from the authors on request.

## Abbreviations

CBM: Chinese Biology Medical Database
CI: Confidence interval
CNKI: Chinese National Knowledge Infrastructure
KPS: Karnofsky performance score
MD: Mean difference
NSCLC: Non-small-cell lung cancer
ORR: Objective tumor response rate
RCTs: Randomized controlled trials
RECIST: Response Evaluation Criteria In Solid Tumors
RR: Risk ratio
TNM: Tumor-node-metastasis
XAPI: Xiao-ai-ping injection
