# Supplementary figures and images for: Xiao-ai-ping injection adjunct with platinum-based chemotherapy for advanced non-small-cell lung cancer: a systematic review and meta-analysis
Source: BMC Complement Med Ther. 2020 Jan 13;20:3. doi: 10.1186/s12906-019-2795-y (PMC7076846; doi:10.1186/s12906-019-2795-y)

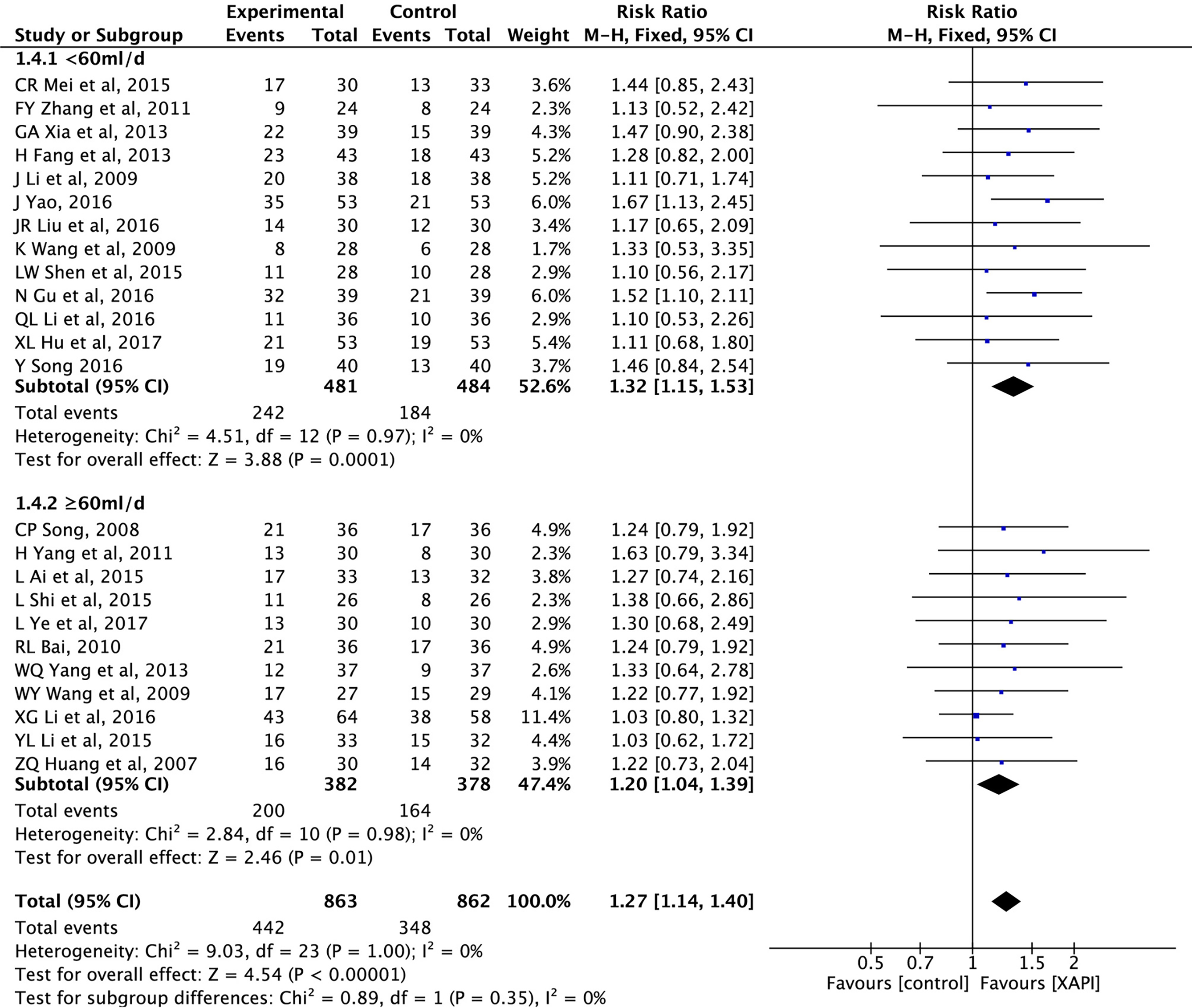

Supplement: Supplementary file 1 — Additional file 1: Figure S1. Subgroup analysis of effects of Xiao-ai-ping-injection (XAPI) on the objective tumor response rate (ORR) in patients with advanced NSCLC according to the different dosages of the analyzed studies. [file 12906_2019_2795_MOESM1_ESM.tif]

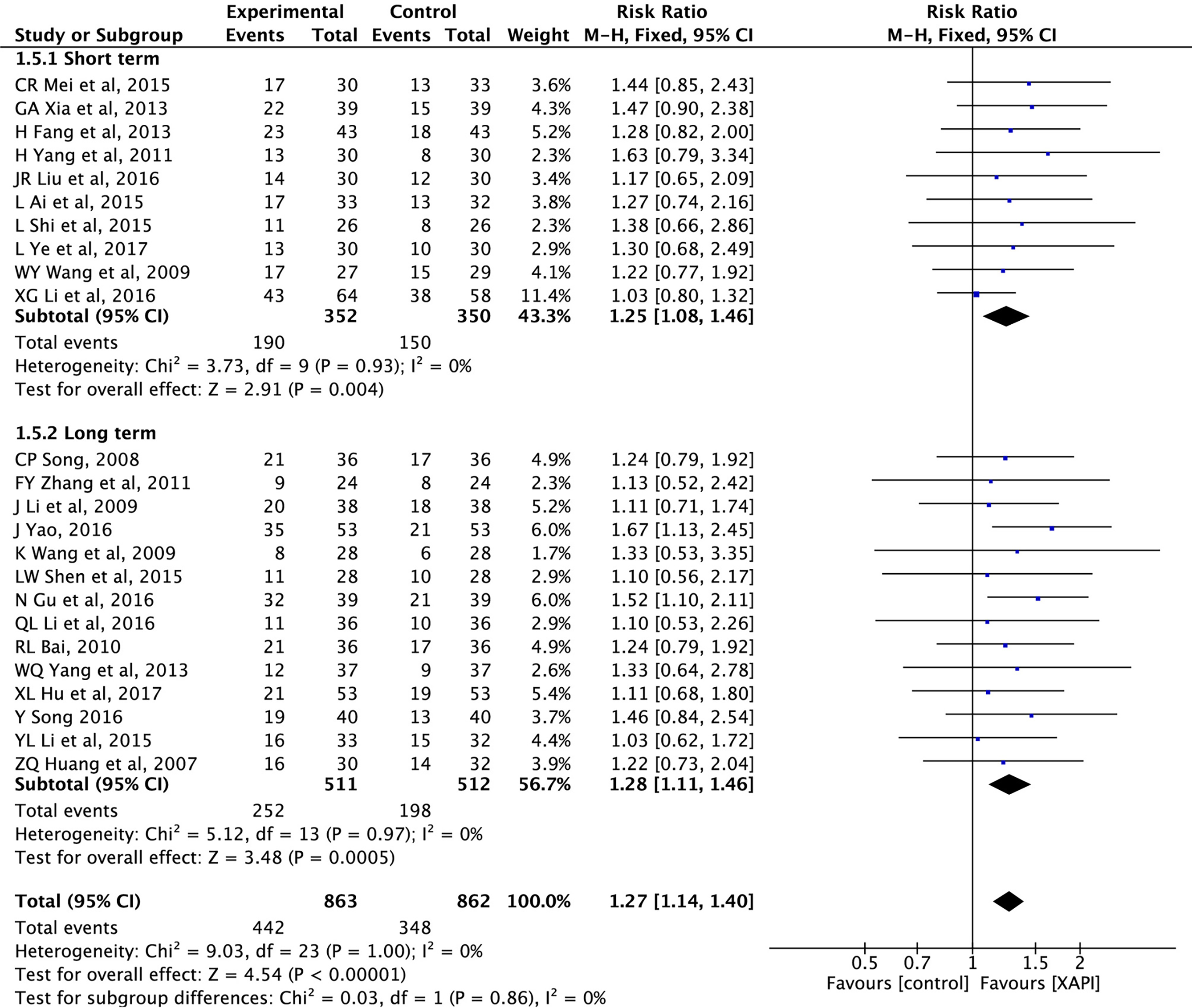

Supplement: Supplementary file 2 — Additional file 2: Figure S2. Subgroup analysis of effects of Xiao-ai-ping-injection (XAPI) on the objective tumor response rate (ORR) in patients with advanced NSCLC according to the treatment duration of the analyzed studies. [file 12906_2019_2795_MOESM2_ESM.tif]

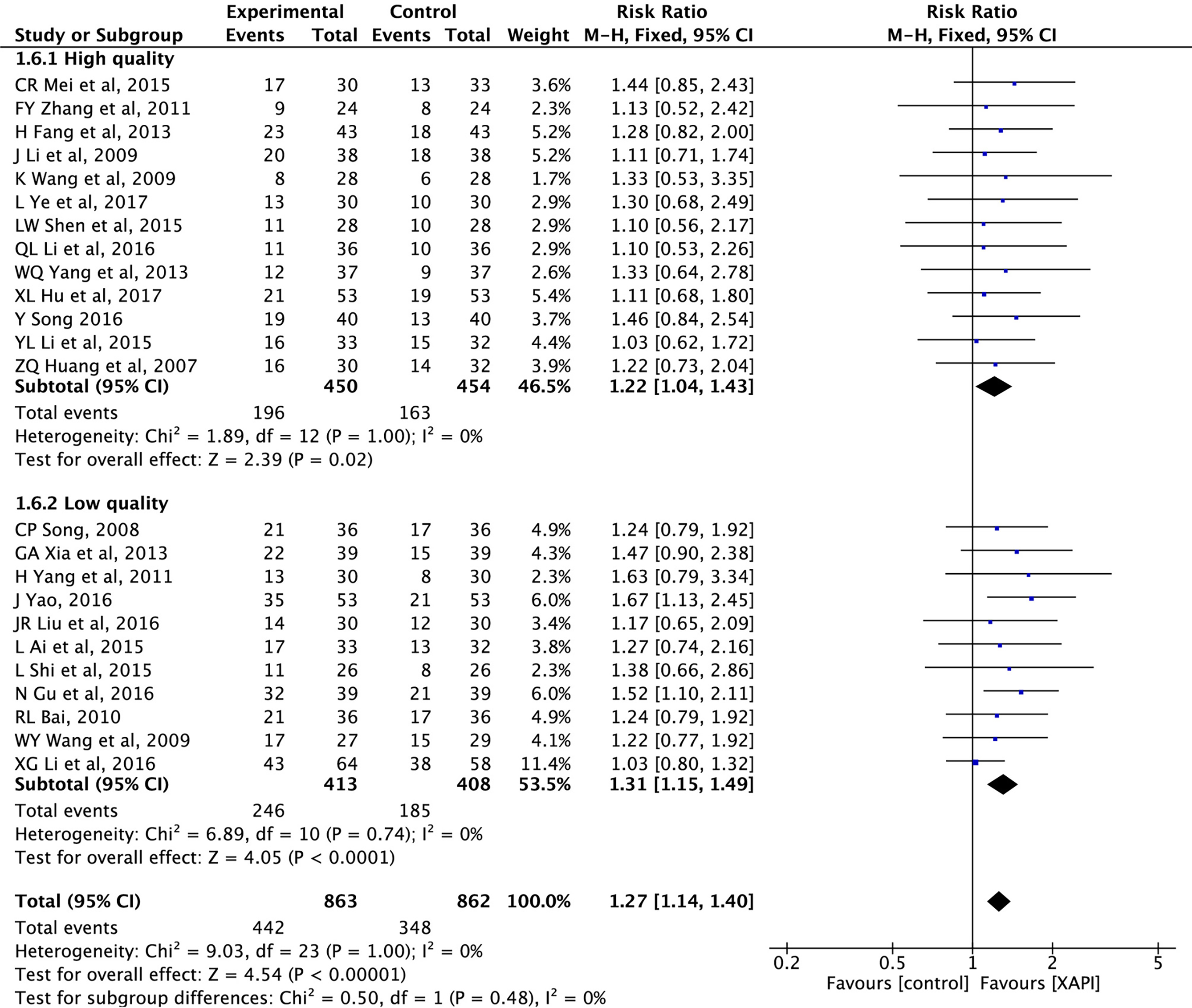

Supplement: Supplementary file 3 — Additional file 3: Figure S3. Subgroup analysis of effects of Xiao-ai-ping-injection (XAPI) on the objective tumor response rate (ORR) in patients with advanced NSCLC according to the methodological quality of the analyzed studies. [file 12906_2019_2795_MOESM3_ESM.tif]

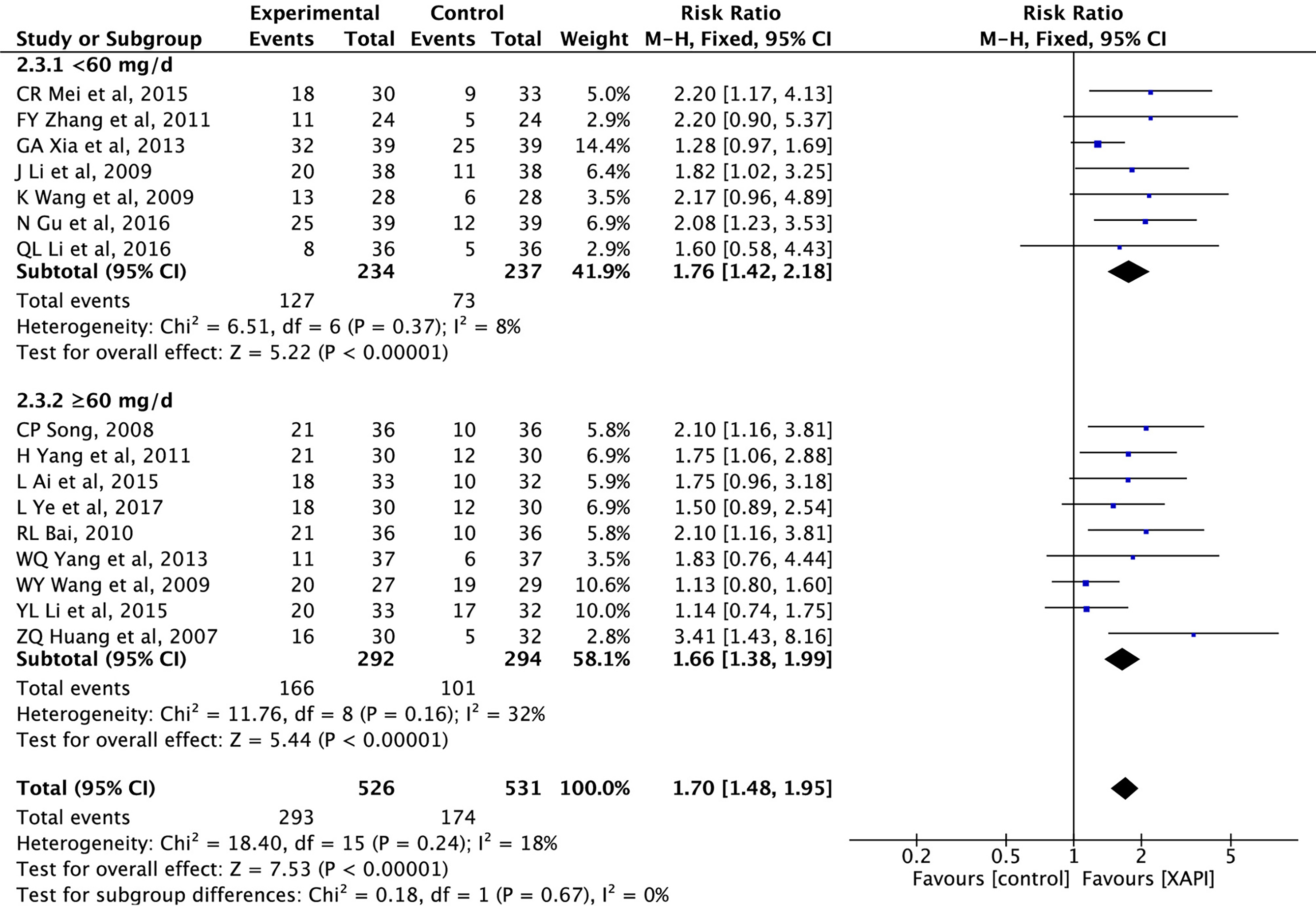

Supplement: Supplementary file 4 — Additional file 4: Figure S4. Subgroup analysis of effects of Xiao-ai-ping-injection (XAPI) on the Karnofsky Performance Status (KPS) in patients with advanced NSCLC according to the different dosages of the analyzed studies. [file 12906_2019_2795_MOESM4_ESM.tif]

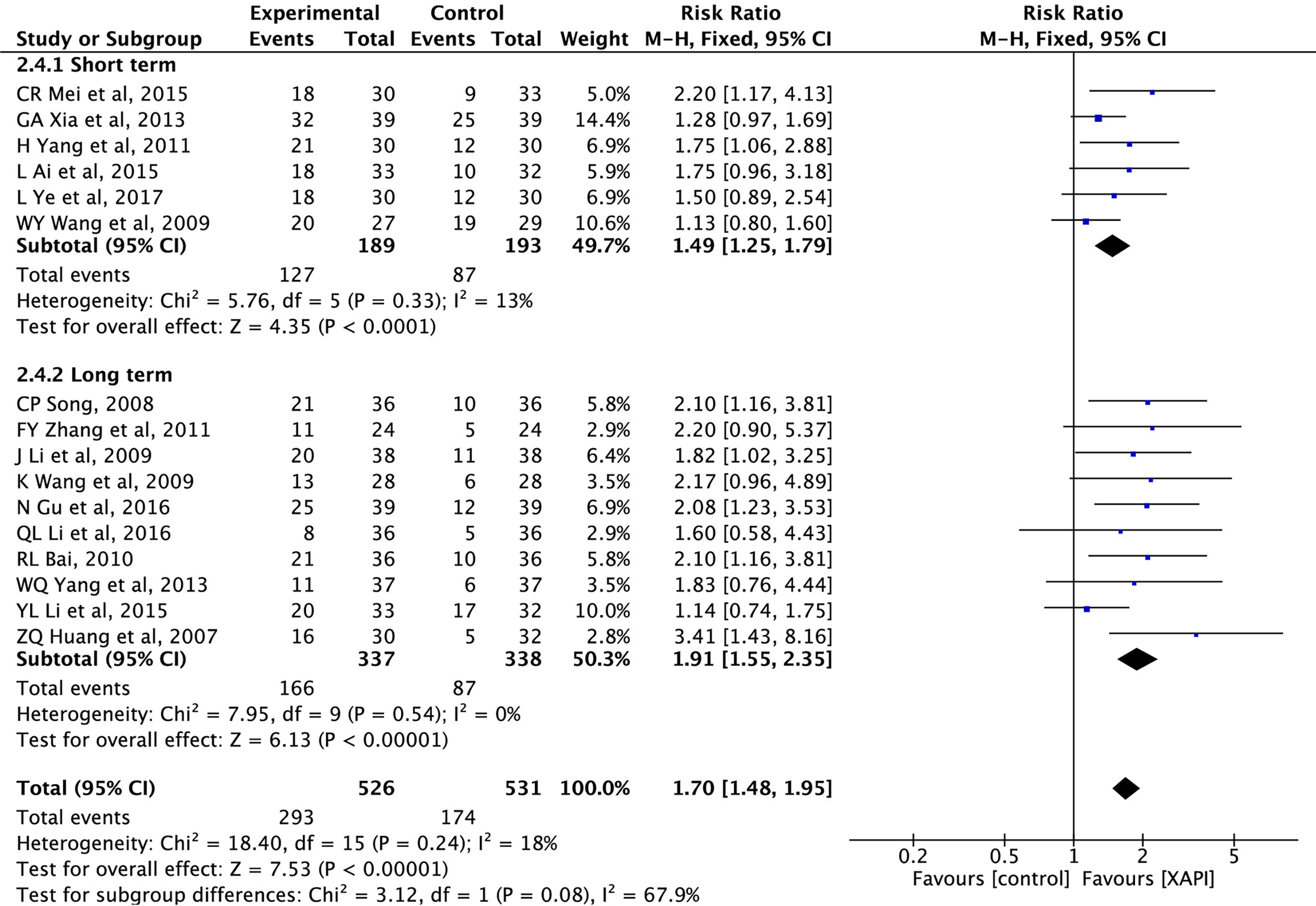

Supplement: Supplementary file 5 — Additional file 5: Figure S5. Subgroup analysis of effects of Xiao-ai-ping-injection (XAPI) on the Karnofsky Performance Status (KPS) in patients with advanced NSCLC according to the treatment duration of the analyzed studies. [file 12906_2019_2795_MOESM5_ESM.tif]

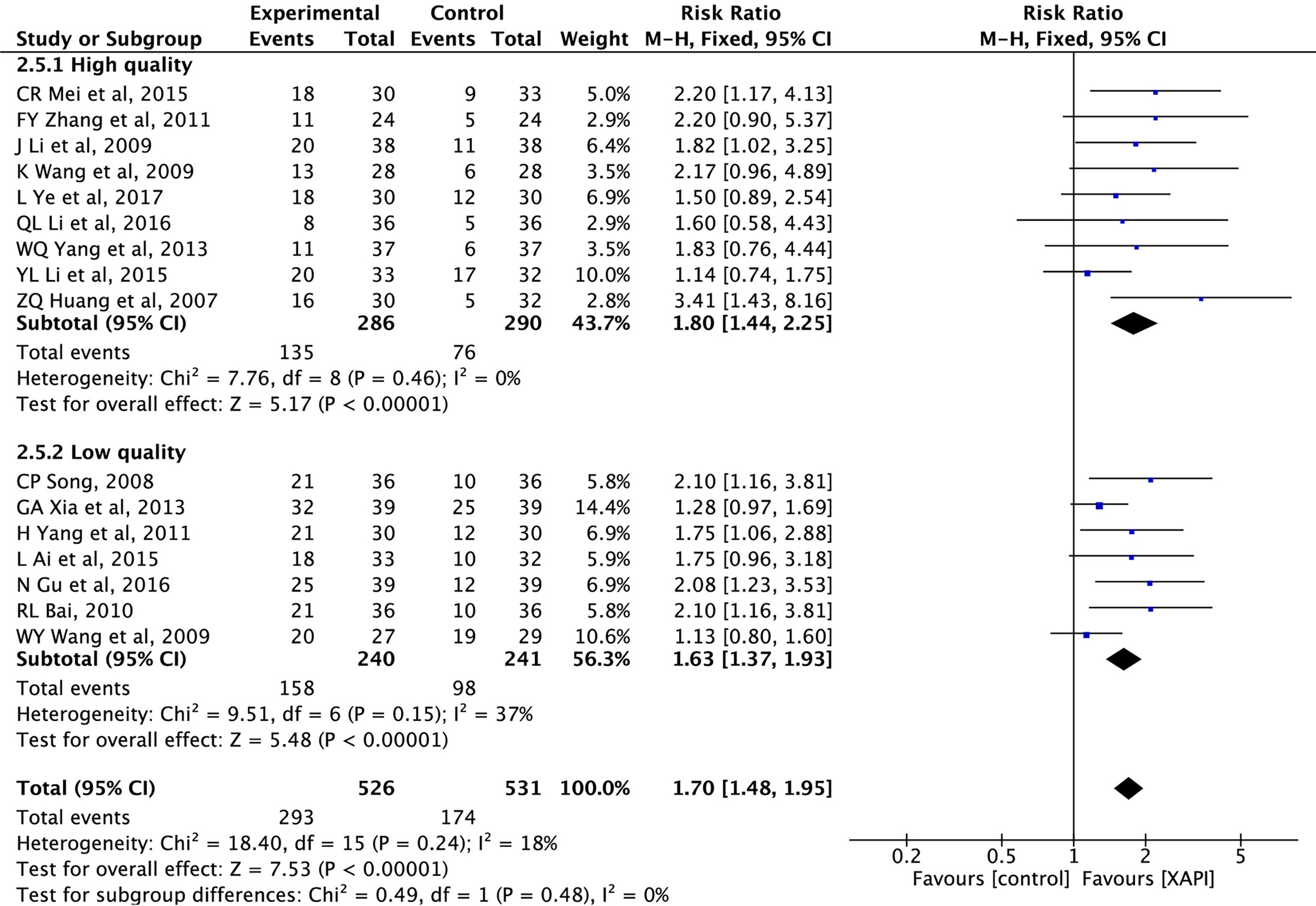

Supplement: Supplementary file 6 — Additional file 6: Figure S6. Subgroup analysis of effects of Xiao-ai-ping-injection (XAPI) on the Karnofsky Performance Status (KPS) in patients with advanced NSCLC according to the methodological quality of the analyzed studies. [file 12906_2019_2795_MOESM6_ESM.tif]
