# Supplementary material for: Xiao-ai-ping injection adjunct with platinum-based chemotherapy for advanced non-small-cell lung cancer: a systematic review and meta-analysis
Source: BMC Complement Med Ther. 2020 Jan 13;20:3. doi: 10.1186/s12906-019-2795-y (PMC7076846; doi:10.1186/s12906-019-2795-y)
Supplement: Supplementary file 7 — Additional file 7: Table S1 Criteria used to assess methodological score. [file 12906_2019_2795_MOESM7_ESM.docx]

**Table S1** Criteria used to assess methodological score

|  | **Score** | | |
| --- | --- | --- | --- |
|  | **0** | **1** | **2** |
| Randomization | … | Not concealed or sure | Concealed randomization |
| Blinding | Not blinded | … | Adjudicators blinded |
| Analysis | Other | … | Intention to treat |
| Patient selection | Selected patients or unable to tell | Consecutive eligible patients | … |
| Comparability of groups at base line | No or not sure | Yes | … |
| Extent of follow-up | < 100% | 100% | … |
| Treatment protocol | Poorly described | Reproducibly described | … |
| Cointerventions | Not described | Described but not equal or not sure | Well described and all  equal |
| Outcomes | Not described | Partially described | Objectively defined |

*The first three questions and last two questions had possible scores of 0, 1 or 2. The middle three questions had possible scores of 0 or 1. The highest possible score was 14. Ellipses indicate data not applicable.
